# Successful Management of Intraoperative Cardiac Arrest in a Patient With Undiagnosed Hypertrophic Cardiomyopathy

**DOI:** 10.7759/cureus.73930

**Published:** 2024-11-18

**Authors:** Nabil Mehdi, Said Khallikane, Bassam Bencharfa, Ayoub Bouchama, Qamouss Youssef

**Affiliations:** 1 Anesthesia and Critical Care, Faculty of Medicine and Pharmacy of Rabat, Rabat, MAR; 2 Anesthesiology and Reanimation, Avicenne Military Hospital, Marrakech, MAR; 3 Anesthesiology, Avicenne Military Hospital, Marrakech, MAR; 4 Anesthesia and Critical Care, Avicenne Military Hospital/Cadi Ayyad University, Marrakech, MAR

**Keywords:** anesthesia complication, anesthesia management, hypertrophic cardiomyopathy, intraoperative cardiac arrest, resuscitation

## Abstract

Intraoperative cardiac arrest presents a significant challenge in surgical settings, particularly in patients with undiagnosed cardiac conditions. This report details the case of a 62-year-old male patient who experienced cardiac arrest during elective laparoscopic cholecystectomy, attributed to previously undiagnosed hypertrophic cardiomyopathy (HCM). The patient exhibited no prior cardiac symptoms and was assessed as low risk preoperatively. Following prompt cardiopulmonary resuscitation (CPR) and defibrillation, return of spontaneous circulation (ROSC) was achieved after approximately 10 minutes. Post-resuscitation, echocardiography confirmed significant left ventricular hypertrophy, leading to a new diagnosis of HCM. This case emphasizes the necessity of thorough cardiovascular evaluation in high-risk surgical patients and outlines effective management strategies for intraoperative emergencies.

## Introduction

This case report discusses the intraoperative cardiac arrest of a 62-year-old male patient undergoing elective laparoscopic cholecystectomy. The cardiac arrest was attributed to undiagnosed hypertrophic cardiomyopathy (HCM), a condition that was not identified during preoperative assessments. HCM, a leading cause of sudden cardiac arrest in individuals under 35, is often asymptomatic and may remain undiagnosed in many patients without prior screening [1]. Although routine preoperative evaluations can help identify cardiovascular risks, undiagnosed cardiac conditions like HCM may not be detected unless specifically sought [2]. This case underscores the importance of comprehensive cardiovascular assessments, particularly in high-risk surgical patients, and highlights the need for effective management strategies during cardiac emergencies in the perioperative setting [3].

## Case presentation

The patient, weighing 100 kg (BMI: 32), had a medical history that included mild hypertension, managed with lisinopril, and type 2 diabetes, treated with metformin. Notably, he had no prior history of cardiac disease or symptoms such as angina, syncope, or dyspnea, suggesting a low risk for perioperative complications. During the preoperative assessment, laboratory tests, including a complete blood count, electrolyte levels, and renal function tests, all returned within normal ranges. An electrocardiogram (ECG) showed normal sinus rhythm without evidence of ischemia or arrhythmias. A thorough physical examination revealed stable vital signs without any significant cardiac abnormalities, and the patient was classified as American Society of Anesthesiologists (ASA) II.

**Figure 1:**
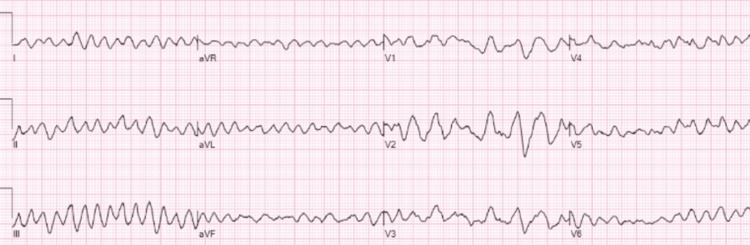
Patient's electrocardiogram showed ventricular fibrillation. aVR: augmented vector right; aVL: augmented vector left; aVF: augmented vector foot

On the day of surgery, anesthesia induction was performed with midazolam (2 mg for sedation), followed by propofol (200 mg for rapid induction) and fentanyl (150 µg for analgesia). During the induction of anesthesia, succinylcholine was administered for neuromuscular blockade, and the patient subsequently developed sudden hypotension and ventricular fibrillation (VF). Succinylcholine is known to be a potential trigger for arrhythmias, especially in patients with underlying cardiac conditions like HCM. However, in this case, it is more likely that the arrhythmia was a result of the patient's undiagnosed HCM, which predisposes individuals to sudden cardiac events. Although succinylcholine may have contributed to the arrhythmia, the primary cause is likely the patient's underlying cardiac condition. In high-risk patients such as this one, consideration of alternative neuromuscular blocking agents, such as rocuronium, might be prudent to minimize the risk of arrhythmias. An ECG monitor was promptly attached, revealing VF with no palpable pulse (Figure 1).

**Figure 2:**
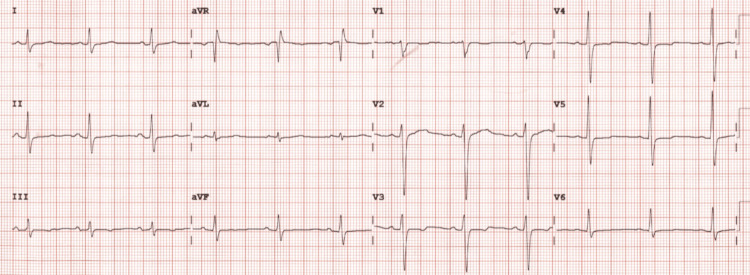
Patient's electrocardiogram showed left ventricular hypertrophy. aVR: augmented vector right; aVL: augmented vector left; aVF: augmented vector foot

During anesthesia induction, the patient's ECG revealed polymorphic ventricular tachycardia (VT), which can be associated with several factors, including electrolyte disturbances such as hypomagnesemia. Hypomagnesemia is a known arrhythmogenic factor and could contribute to the development of VT in susceptible individuals. However, given the patient's undiagnosed HCM, it is more likely that the VT was a manifestation of HCM rather than hypomagnesemia alone. Magnesium levels were not immediately assessed during the acute phase of the event, but further investigation into electrolyte disturbances could be valuable. Immediate cardiopulmonary resuscitation (CPR) was initiated, and an automated external defibrillator (AED) was used to deliver a shock within one minute of cardiac arrest. Following several rounds of CPR and medication administration, including epinephrine and amiodarone, return of spontaneous circulation (ROSC) was achieved after approximately 10 minutes. The patient's blood pressure stabilized at 110/70 mmHg, and the heart rhythm reverted to normal sinus rhythm. After stabilization, the patient was transferred to the intensive care unit (ICU) for further evaluation and management. In the ICU, the patient underwent continuous cardiac monitoring to detect any potential arrhythmias. An echocardiogram was performed post-resuscitation, revealing significant left ventricular hypertrophy consistent with HCM, which had remained undiagnosed prior to surgery (Figure 2).

Additionally, there was evidence of left ventricular outflow tract obstruction (LVOTO), which is common in HCM, and systolic anterior motion (SAM) of the mitral valve. The left atrium (LA) was mildly dilated, and the ejection fraction (EF) was preserved at 55%, indicating adequate left ventricular systolic function despite the hypertrophic changes.

Neuroprotective measures, including therapeutic hypothermia, were initiated to minimize potential neurological damage due to prolonged cardiac arrest [4]. A cardiology consultation was sought for further evaluation and management of the newly diagnosed HCM, leading to the initiation of beta-blocker therapy and patient education regarding the condition [5].

## Discussion

Pathophysiology of HCM

HCM is a genetic condition that often remains asymptomatic until a significant event, such as the intraoperative cardiac arrest observed in this patient [6,7]. In this case, the undiagnosed HCM led to a sudden event during anesthesia induction. While many patients with HCM remain asymptomatic, especially in the early stages, routine screening for cardiac conditions is essential, particularly for patients with risk factors such as obesity, family history of cardiovascular disease, and unexplained syncope [8,9].

This case underscores the critical importance of identifying cardiovascular risks in patients undergoing surgery. HCM is often asymptomatic, particularly in the early stages, which complicates preoperative assessments [10]. Patients with HCM may experience sudden cardiac events, particularly during times of physiological stress, such as anesthesia and surgery [11].

In this case, the patient's undiagnosed HCM led to an unexpected intraoperative cardiac arrest. Literature suggests that up to 20% of patients with HCM may present with sudden cardiac death as their first clinical manifestation [12]. Therefore, enhanced screening protocols, including echocardiography, should be considered for patients with risk factors such as obesity, family history of cardiac disease, and unexplained syncope [13].

The management of intraoperative cardiac arrest requires prompt and effective intervention. The American Heart Association emphasizes the importance of high-quality CPR and early defibrillation in improving survival outcomes [14]. In this case, the rapid response by the anesthetic team, including the use of an AED, was critical in achieving ROSC.

Moreover, postoperative care in the ICU should include vigilant monitoring for arrhythmias, especially in patients with undiagnosed cardiomyopathy. Initiating appropriate medical therapy, such as beta-blockers, can significantly improve patient outcomes and reduce the risk of future cardiac events [15]. Education for patients and families about the implications of HCM is equally important in ensuring adherence to follow-up care and lifestyle modifications.

## Conclusions

This case illustrates the importance of thorough preoperative cardiovascular evaluation, especially in patients with subtle or undiagnosed risk factors like HCM. The patient's unexpected intraoperative cardiac arrest was managed successfully with immediate CPR and defibrillation. Early identification of HCM through comprehensive screening may prevent similar adverse events. A multi-disciplinary approach involving anesthesiologists, surgeons, and cardiologists is essential for managing high-risk surgical patients.
